# One-year prevalence and clinical characteristics in chronic dizziness: The 2019–2020 Korean National Health and Nutrition Examination Survey

**DOI:** 10.3389/fneur.2022.1016718

**Published:** 2022-12-01

**Authors:** Eun Ji Kim, Hee-Jung Song, Hak In Lee, Eunjin Kwon, Seong-Hae Jeong

**Affiliations:** ^1^Department of Neurology, Chungnam National University Hospital, Daejeon, South Korea; ^2^Department of Neurology, Chungnam National University Sejong Hospital, Sejong, South Korea; ^3^Department of Neurology, Chungnam National University School of Medicine, Daejeon, South Korea

**Keywords:** prevalence, chronic, dizziness, imbalance, obesity

## Abstract

**Introduction:**

In this cross-sectional study, we investigated the 1-year prevalence and related factors in the general population with an experience of chronic dizziness.

**Methods:**

This study analyzed persons (*n* = 5,163) who respond to dizziness and nutrition questionnaire from participant of Korean National Health and Nutrition Examination Survey (KNHANES, 2019-2020).

**Results:**

Of individuals over 40 years, 25.3% of the general population (61.6% females) reported either dizziness or imbalance for the past year. Moreover, 4.8% of the patients reported they suffered from chronic dizziness or imbalance for more than 3 months. In multiple regression analysis, patients with chronic dizziness were older, females, had lower body mass index (BMI), had stress awareness, and had a history of tinnitus within 1 year (>5 min per episode). Relative to normal body weight, both overweight and mild obesity (obesity stages 1 and 2) were associated with a significantly lower risk of chronic dizziness. Overweight, obesity stage 1, and obesity stage 2 had odds ratios of 0.549 [95% confidence interval (CI), 0.332–0.910], 0.445 (95% CI, 0.273–0.727), and 0.234 (95% CI, 0.070–0.779), respectively.

**Conclusions:**

In this study, the prevalence of chronic dizziness in the general population was 4.8%. Our study demonstrated that overweight and mild obesity were independently associated with a lower risk of chronic dizziness in adults for the past year. Therefore, the optimal BMI for patients with dizziness should be defined and managed according to an integrated care pathway.

## Introduction

Vertigo and dizziness rank among the 10 most common reasons for referral to neurologists in the emergency room and daily clinical practice (1). Dizziness and imbalance may include multifactorial deficits in peripheral and central sensory functions (visual, vestibular, and somatosensory), musculoskeletal insufficiencies (sarcopenia and arthritis), deficits in cognitive postural control, and emotions (anxiety and depression) (2). Dizziness and imbalance can be chronic, which causes a decrement in quality of life, increased mortality, socio-economic loss, and a higher risk of falls (3–6). Chronicity is a significant clinical feature of several diseases that should be analyzed because those with chronic features or symptoms can have poorer outcomes. Despite this concern, few studies have gauged the general population's prevalence and associated epidemiological factors for chronic dizziness (7, 8). Even the strength of the association between dizziness and imbalance, the accompanied frequency of standing, walking difficulty, or falls is also unclear. The Korean National Health and Nutrition Examination Survey (KNHANES) is a large-scale, highly powered survey (aged ≥ 40 years) that included chronic dizziness between 2019 and 2020. This survey defined chronic dizziness as one or >3 months in a recent year (9). Herein, we investigated the weighted prevalence, clinical characteristics, and associated factors of chronic dizziness in the Korean adult population using the 2019–2020 KNHANES.

## Methods

### Study population and data collection

**Figure 1:**
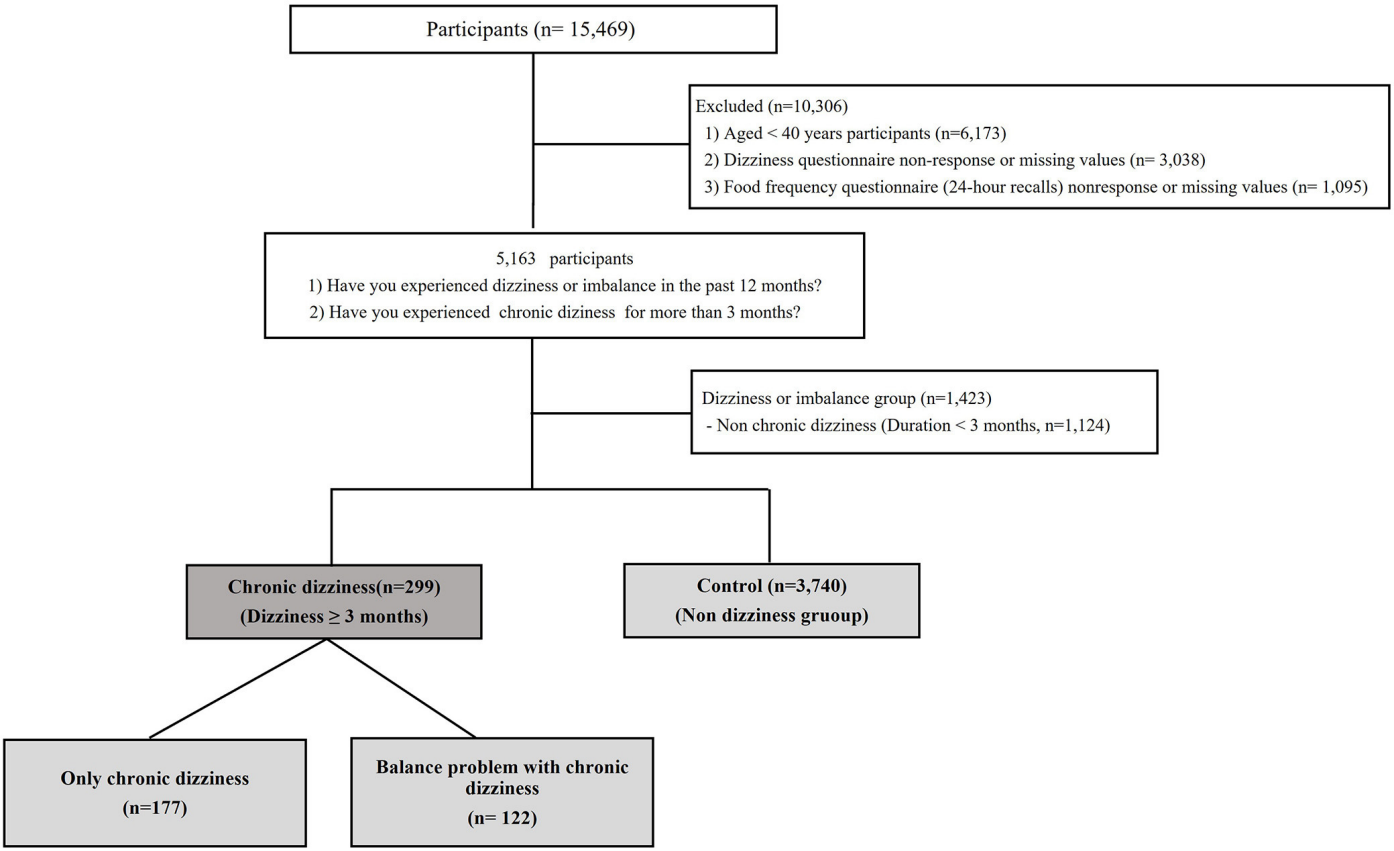
Flow chart in this study.

From the 2019–2020 KNHANES database, we selected 5,163 participants who met the following criteria: (1) response to the dizziness questionnaire and (2) response to the health questionnaire, including food frequency (Figure 1). Since 1998, the KNHANES has been conducted periodically to assess the health and nutritional status of the civilian, non-institutionalized population of South Korea. Participants were selected using proportional allocation systemic sampling with multistage stratification (10). Balance problem and dizziness were assessed using the following question: “during the past 12 months, have you had dizziness or difficulty with balance?”. Furthermore, participants were asked whether they had experienced chronic dizziness (“In the past 3 months or more, have you felt chronically dizzy?”), postural instability in standing or walking (“In the past 3 months or more, have you had difficulty maintaining the standing position or walking?”), and falling (“In the past 3 months or more, have you experienced falling?”) for more than 3 months in the recent year (Table 1). A control group was defined as those who had not experienced dizziness in the past year.

**Table 1:** Questionnaire about chronic dizziness/imbalance.

**Items**	**Yes**	**No**
In the past 12 months, have you experienced dizziness or loss of balance?		
In the past 3 months or more, have you felt chronically dizzy?		
For the past 3 months or more, have you found it difficult to maintain a standing position?		
For the past 3 months or more, have you had difficulty walking?		
In the past 3 months or more, have you experienced falling?		

Data collection included fundamental demographic factors, laboratory data, and other conditions that could cause dizziness or imbalance. The definition of the selected variables was determined according to the KNHANES guidelines (9). Age, sex, and house income were selected as demographic data. BMI was calculated as weight in kilograms (Body composition analyzer, InBody 970^®^, Korea) divided by height in meters squared (Digital mobile stadiometer, seca274^®^, Germany). Perceived stress, hypertension, diabetes mellitus, dyslipidemia, anemia, stroke, kidney disease, depression, tinnitus, hearing impairment, and tympanic abnormality were comorbid conditions determined using laboratory data and questionnaires. Moreover, we evaluated health behaviors, including smoking, alcohol drinking, and weight change, in 1 year using a questionnaire. Additionally, the nutrition survey was analyzed (9).

#### Household income

The household income level was classified into four groups (low, middle lower, upper middle, and high) according to the income quartile. In this study, the middle lower and upper middle levels were combined as medium; subsequently, the household income level was reclassified into three groups (high, medium, and low) (9).

#### BMI

To facilitate standardized comparisons with other analyses, we calculated odds ratios (ORs) for chronic dizziness in the six World Health Organization (WHO)-defined baseline BMI categories: underweight (BMI < 18.5 kg/m^2^), normal (18.5 ≤ BMI < 23 kg/m^2^), overweight (23 ≤ BMI < 25 kg/m^2^), obesity stage 1 (25 ≤ BMI < 30 kg/m^2^), obesity stage 2 (30 ≤ BMI < 35 kg/m^2^), and obesity stage 3 (BMI ≥ 35 kg/m^2^), according to the WHO Asia–Pacific guidelines (11).

#### Comorbid conditions

Perceived stress is the feelings or thoughts an individual has about how much stress they are under daily. The stress level was classified into four groups (rare, mild, moderate, and severe). In this study, we classified the participants into two groups as less stress (rare and mild) and those who experienced much stress (moderate and severe) (9). Hypertension was defined as systolic blood pressure ≥ 140 mmHg, diastolic blood pressure ≥ 90 mmHg, or taking antihypertensive medications irrespective of blood pressure. Diabetes was defined as fasting blood sugar ≥ 126 mg/dL or diagnosed by a doctor or taking a hypoglycemic agent, insulin injection, or glycated hemoglobin concentration ≥ 6.5%. Hypercholesterolemia was defined as blood total cholesterol level ≥ 240 mg/dL or taking cholesterol-lowering drugs. Hypertriglyceridemia was defined as a blood triglyceride concentration ≥ 200 mg/dL. Low-density lipoprotein cholesterol and high-density lipoprotein cholesterol were classified as a cut point of 160 and 40 mg/dL, respectively. Anemia was defined as blood hemoglobin <12 and <13 mg/dL in females and males, respectively. Patients with the disease (stroke, kidney disease, and depression disorder) were diagnosed with the disease by a physician. Coexistence of tinnitus was defined as ringing in own ears (Jijik, beep, hum, and machine sound) for more than 5 min in the past 12 months (9). Mild hearing impairment was defined as a pure unaided tone hearing threshold for the prominent ear of 26–41 decibels (dB) and average hearing threshold levels (HLs) for the frequencies of 0.5, 1.0, 2.0, and 4.0 kHz. Moderate to profound hearing impairment was defined as pure unaided tone HL for the superior level of 41 dB or greater and HLs for the frequencies of 0.5, 1.0, 2.0, and 4.0 kHz. In this study, hearing impairment was defined as mild to profound hearing impairment. In the 2019–2020 KNHANES, air conduction pure-tone thresholds were obtained in a soundproof booth using an automatic audiometer (AD629; Interacoustics, Denmark) (9).

#### Health behaviors

Alcohol consumption was assessed by asking the participants about their drinking behavior for the past year before the interview. During the interview, alcohol consumption was recategorized as none, <1 per month, 1–4 per month (1 and 2–4 per month), and ≥2 per week (2–3 and >4 per week). Smoking was classified into two groups (never smoker or ex-smoker and current smoker) (9).

#### Nutrition

The nutrition survey of the KNHANES comprised a survey of dietary habits, a 1-day 24 h recall, and a food frequency questionnaire. After the health interview and examination, trained dietitians collected the nutrition survey data in the participants' homes. Daily energy intake was calculated using the Korean Foods and Nutrients Database of the Rural Development Administration. The analysis included the following items: carbohydrate, protein, and fat intakes. Furthermore, the following items were analyzed: minerals (Ca, P, Fe, Na, and K), vitamins (Vitamin A, B1, B2, B3, C, folic acid, and ß carotene), water, and dietary fiber (Appendix). Vitamin D levels could not be analyzed because KNHANES 2019–2020 did not test for serum vitamin D. We used the criteria of recommended nutrient intake and adequate intake as well as tolerable upper intake level to facilitate standardized comparisons with other analyzes (9).

### Ethics statement

The KNHANES was approved by the Institutional Review Board (IRB) of the Korean Centers for Disease Control and Prevention (KCDC) (IRB: 2018-01-03-C-A, 2018-01-03-2C-A). However, approval from the IRB to perform this study was not required for the following reasons: (1) The KNHANES data do not include personal information, and each individual was assigned a unique personal identification number. (2) The KNHANES is open to the public and can be accessed using the following link: https://knhanes.cdc.go.kr.

### Statistical analysis

The KNHANES participants were not randomly sampled. The survey used a complex, stratified, multistage probability-sampling model; thus, individual participants were not equally representative of the Korean population. Therefore, in obtaining representative prevalence rates from the dataset, the consideration of the power of each participant (sample weight) as representative of the Korean population was necessary. Following approval from the KCDC, we received a survey dataset that included information regarding the survey location, strata by age, sex, and the sample weight for each participant. To provide representative estimates of the non-institutionalized Korean civilian population, the survey sample weights, which were calculated by considering the sampling rate, response rate, and age/sex proportions of the reference population (2005 Korean National Census Registry), were used in all analyzes. We analyzed the weighted prevalence of chronic dizziness and compared clinical characteristics by the presence of chronic dizziness using the chi-square test and *t*-test. Potential associated factors were evaluated and selected for univariate analysis using a logistic regression model. Clinically essential variables with a *p* < 0.05 were selected for multivariate analysis using a logistic regression model. Variables with multicollinearity issues were not included in the logistic regression model. Statistical analyses were performed using statistical package for the social sciences (SPSS) Complex Samples analysis (ver. 21.0 for Windows; SPSS, Chicago, IL, USA).

## Results

### Prevalence of chronic dizziness

A total of 15,469 individuals were enrolled. Finally, a total of 5,163 individuals were analyzed, of whom 1,423 (27.6%) indicated dizziness or imbalance and 3,740 (72.4%) did not experience dizziness or imbalance over the past year (Table 2). Of the patients with dizziness, chronic dizziness (dizziness ≥ 3 months) was observed in 299 (5.8%) individuals. The study population's weighted demographics and clinical characteristics are demonstrated in Tables 2, 3. In patients with chronic dizziness, 40.8% (122/299) had trouble with postural control, including standing, walking, and falling, and other patients (59.2%, 177/299) complained only of dizziness without experiencing postural instability. Overall, the 1-year weighted prevalence of chronic dizziness was 4.8% (Table 2).

**Table 2:** Estimated prevalence of chronic dizziness in the Korean adult population.

**Classification**	**Unweighted number (*n* = 5,163)**	**Weighted prevalence, % [95%CI] (*n* = 27,767,663)**
**Chronic dizziness** **(Duration** **≥3 months)**	299	4.8 [4.1–5.6]
Sole dizziness	177	3.0 [2.4–3.6]
Balance problem	122	1.8 [1.4–2.4]
Only falling	20	0.3 [0.2–0.5]
Only walking difficulty	7	0.1 [0.0–0.1]
Only standing difficulty	17	0.3 [0.1–0.5]
Falling and walking difficulty	2	0.0 [0.0–0.2]
Falling and standing difficulty	5	0.1 [0.0–0.2]
Walking and standing difficulty	45	0.7 [0.5–1.1]
All of them % (*n*)^*^	26	0.4 [0.2–0.6]

* Falling, and difficulty of walking and standing.

**Table 3:** Clinical characteristics by the presence of chronic dizziness.

Variable^*^	**Chronic dizziness (*n* = 299)**	**No dizziness (*n* = 3,740)**	***P*-value**
**Demographic data**			
Age (mean)	299 (64.5)	3,740 (57.4)	**<** **0.001**
Sex (%)			**<** **0.001**
Men	84 (3.7)	1,682 (96.3)	
Female	215 (8.3)	2,058 (91.7)	
Household income (%)			**<** **0.001**
Upper	40 (3.4)	1,071 (96.6)	
Middle	122 (5.0)	1,880 (95.0)	
Lower	137 (13.7)	777 (86.3)	
**Laboratory data**			
BMI (kg/m^2^) (mean)	293 (23.5)	3,695 (24.3)	**0.003**
Obesity (%)			**<** **0.001**
Underweight (BMI < 18.5)	15 (11.3)	91 (88.7)	
Normal (18.5 ≤ BMI < 23)	127 (8.3)	1,275 (91.7)	
Pre-obesity (overweight) (23 ≤ BMI < 25)	59 (4.5)	914 (95.5)	
Obesity stage 1 (25 ≤ BMI < 30)	77 (4.4)	1,233 (95.6)	
Obesity stage 2 (30 ≤ BMI < 35)	12 (3.9)	164 (96.1)	
Obesity stage 3 (BMI ≥ 35)	3 (14.3)	18 (85.7)	
**Comorbid conditions (laboratory test, questionnaire)**		
Perceived stress (%)			**<** **0.001**
Low	185 (4.5)	2,984 (95.5)	
High	111 (11.5)	724 (88.5)	
Hypertension (%)			0.517
Normal	92 (6.4)	1,185 (93.6)	
Prehypertension, hypertension	204 (5.8)	2,533 (94.2)	
Diabetes mellitus (%)			0.445
Normal	87 (6.3)	1,101 (93.7)	
Pre-diabetes, diabetes	190 (5.6)	2,473 (94.4)	
Hypercholesterolemia (%)			0.453
No	181 (5.6)	2,470 (94.4)	
Yes (≥240 mg/dL)	95 (6.2)	1,105 (93.8)	
Hypertriglyceridemia (%)			**0.008**
No	225 (6.6)	2,638 (93.4)	
Yes (≥200)	23 (3.6)	437 (96.4)	
Hyper-LDL cholesterolemia (%)			0.066
No	266 (6.1)	3,332 (93.9)	
Yes (≥160)	20 (3.9)	349 (96.1)	
Hypo-HDL cholesterolemia (%)			0.849
No	226 (5.9)	3,056 (94.1)	
Yes (< 40)	60 (6.0)	625 (94.0)	
Anemia (%)			**<** **0.001**
No	219 (5.3)	3,218 (94.7)	
Yes	67 (10.0)	463 (90.0)	
Diagnosis of stroke (%)			**<** **0.001**
No	233 (5.2)	3,384 (94.8)	
Yes	21 (12.6)	93 (87.4)	
Diagnosis of kidney disease (%)			0.316
No	249 (5.4)	3,405 (94.6)	
Yes	4 (3.0)	66 (97.0)	
Diagnosis of depression (%)			**<** **0.001**
No	225 (5.1)	3,319 (94.9)	
Yes	28 (12.9)	152 (87.1)	
Tinnitus (≥ 5 min, within 1 year) (%)			**<** **0.001**
No	211 (4.8)	3,379 (95.2)	
Yes	84 (17.1)	345 (82.9)	
Hearing impairment (%)			**<** **0.001**
No	94 (3.9)	1,923 (96.1)	
Mild	60 (6.2)	771 (93.8)	
Moderate or greater	92 (12.6)	556 (87.4)	
Tympanic membrane abnormality (%)			0.075
No	267 (5.9)	3,460 (94.1)	
Yes	31 (8.3)	260 (91.7)	
**Health behavior(questionnaire)**			
Smoking status (%)			0.191
Never smoker, ex-smoker	259 (6.3)	3,154 (93.7)	
Current smoker	37 (4.8)	559 (95.2)	
Secondhand smoke exposure (%)			0.493
Yes	35 (4.3)	613 (95.7)	
No	76(3.7)	1,742 (96.3)	
Alcohol drinking (%)			**0.002**
none	78 (7.6)	776 (92.4)	
< 1 times/month	55 (6.3)	654 (93.7)	
≥ 1~4 times/month	43 (3.8)	975 (96.2)	
≥ 2 times/week	42 (4.1)	773 (95.9)	
Weight change in 1 year (%)			0.146
No change	186 (5.7)	2,559 (94.3)	
Weight loss	58 (8.0)	465 (92.0)	
Weight gain	52 (5.7)	689 (94.3)	

BMI, body mass index.

* Each value is expressed in the form of unweighted numbers (weighted %) except age (mean) and BMI (mean).

Statistically significant variables are indicated in bold.

### Associated factors

Chronic dizziness was defined as dizziness lasting for 3 months or longer. In this study, 3,740 individuals who did not experience dizziness or imbalance over the past year were used as the control group. All statistical tests were performed in chronic dizziness and control group. The average age of participants with chronic dizziness was higher than that of the control group (64.5 ± 0.9 vs. 57.4 ± 0.3 years, respectively, *p* < 0.001); moreover, the percentage of females was higher (68.8 ± 3.1% vs. 48.6 ± 0.8% in the chronic dizziness and control groups, respectively, *p* < 0.001, Table 3). Chronic dizziness was observed to significantly increase with age, that is, above the age of 40. Significant differences in house income, BMI, perceived stress, hypertriglyceridemia, anemia, diagnosis of stroke and depression, alcohol drinking, tinnitus, and hearing impairment were noted between the chronic dizziness and control groups (Table 3).

The only chronic dizziness group had a lower BMI than the control group (mean ± standard error; 23.1 ± 0.3 vs. 24.3 ± 0.1, respectively, *p* < 0.001). However, no difference in BMI between the balance problems with chronic dizziness and control groups was observed. Among the variables significantly associated with chronic dizziness in univariate analysis, age, sex, BMI, hypertriglyceridemia, stroke, alcohol history, household income, perceived stress, anemia, and tinnitus were selected for the multivariable analysis (Table 4). Variables, including hearing impairment, history of depressive disorder, and nutrition with multicollinearity problems, were excluded from the logistic regression model. In the multivariable analysis, age [*p* = 0.003, OR = 1.034, 95% confidence interval (CI), 1.011–1.057], female sex (*p* = 0.006, OR = 1.861, 95% CI, 1.200–2.887), overweight (*p* = 0.020, OR = 0.549, 95% CI, 0.332–0.910), obesity stage 1 (*p* = 0.001, OR = 0.445, 95% CI, 0.273–0.727), obesity stage 2 (*p* = 0.018, OR = 0.234, 95% CI, 0.070–0.779), high stress (*p* < 0.001, OR = 3.726, 95% CI, 2.560–5.423), and tinnitus of >5 min within 1 year (*p* < 0.001, OR = 4.598; 95% CI, 2.935–7.205) remained as the independent factors associated with dizziness. The differences in the ORs of the BMI classification between the only chronic dizziness and balance problems with chronic dizziness groups, using the normal BMI (18.5–23.0 kg/m^2^) as the reference, are presented in Tables 5, 6. As observed in Table 5, in the only chronic dizziness group, the protective effect of overweight and obesity stage 1 was significant, wherein the effect remained statistically significant in both models 1 (age and sex) and 2 (age, sex, tinnitus, and perceived stress). In the crude model, participants with balance disturbance and chronic dizziness had lower odds in the overweight group (Table 6). After adjusting for age and sex in model 1, participants with obese stage 3 had higher odds of balance problems in addition to chronic dizziness, whereas participants with overweight had lower odds than those with normal BMI. However, the overweight protective effect did not remain in model 2 (Table 6).

**Table 4:** Multiple logistic regression analysis for chronic dizziness.

**Variable**	**Multivariable logistic regression analysis**		
	**Odds ratio**	**95% CI**	***P*-value**
Age	1.034	1.011–1.057	**0.003**
Sex			**0.006**
Men	Reference		
Women	1.861	1.200–2.887	**0.006**
Household income			0.090
Upper	Reference		
Middle	1.103	0.640–1.900	0.724
Lower	1.907	0.995–3.653	0.052
Obesity			**0.007**
Normal (18.5 ≤ BMI < 23)	Reference		
Underweight (BMI < 18.5)	0.924	0.305–2.797	0.889
Overweight (23 ≤ BMI < 25)	0.549	0.332–0.910	**0.020**
Obesity stage 1 (25 ≤ BMI < 30)	0.445	0.273–0.727	**0.001**
Obesity stage 2 (30 ≤ BMI < 35)	0.234	0.070–0.779	**0.018**
Obesity stage 3 (BMI ≥ 35)	1.221	0.238–6.275	0.810
Perceived stress			**<** **0.001**
Low	Reference		
High	3.726	2.560–5.423	**<** **0.001**
Hypertriglyceridemia			0.606
No	Reference		
Yes (≥200)	0.851	0.460–1.575	0.606
Anemia			0.145
No	Reference		
Yes	1.510	0.867–2.629	0.145
Stroke			0.179
No	Reference		
Yes	1.838	0.755–4.474	0.179
Tinnitus (≥ 5 min, within 1 year)			**<** **0.001**
No	Reference		
Yes	4.598	2.935–7.205	**<** **0.001**
Alcohol drinking			0.323
None	Reference		
< 1 times/month	1.042	0.615–1.768	0.877
≥ 1~4 times/month	0.664	0.380–1.161	0.150
≥ 2 times/week	0.999	0.560–1.781	0.997

Statistically significant variables are indicated in bold.

**Table 5:** Logistic regression analysis for chronic dizziness according to the body mass index.

	**Only chronic dizziness**								
**Variable**	**Crude (unadjusted)**			**Model 1**			**Model 2**		
	**Odds**			**Odds**			**Odds**		
	**ratio**	**95% CI**	***P*-value**	**ratio**	**95% CI**	***P*-value**	**ratio**	**95% CI**	***P*-value**
Obesity									
Normal (18.5 ≤ BMI < 23)	Reference			Reference			Reference		
Underweight (BMI < 18.5)	1.696	0.775–3.713	0.186	1.632	0.738–3.610	0.225	1.752	0.767–4.001	0.182
**Overweight (23** **≤BMI** ** < 25)**	**0.508**	0.316–0.814	**0.005**	**0.515**	0.318–0.835	**0.007**	**0.509**	0.308–0.840	**0.008**
**Obesity stage 1 (25** **≤BMI** ** < 30)**	**0.406**	0.235–0.653	**<** **0.001**	**0.431**	0.269–0.689	**<** **0.001**	**0.462**	0.290–0.735	**0.001**
Obesity stage 2 (30 ≤ BMI < 35)	0.473	0.186–1.202	0.115	0.564	0.218–1.461	0.237	0.578	0.215–1.555	0.276
Obesity stage 3 (BMI ≥ 35)	0.307	0.039–2.428	0.261	0.387	0.047–3.180	0.376	0.341	0.041–2.867	0.321

Model 1: adjusted sex, age.

Model 2: adjusted sex, age, tinnitus, perceived stress.

Reference category(group) : no dizziness (control).

BMI, body mass index.

Statistically significant variables are indicated in bold.

**Table 6:** Logistic regression analysis for balance problem with chronic dizziness according to the body mass index.

	**Balance problem with chronic dizziness**								
**Variable**	**Crude (unadjusted)**			**Model 1**			**Model 2**		
	**Odds**			**Odds**			**Odds**		
	**ratio**	**95% CI**	***P*-value**	**ratio**	**95% CI**	***P*-value**	**ratio**	**95% CI**	***P*-value**
Obesity									
Normal (18.5 ≤ BMI < 23)	Reference						Reference		
Underweight (BMI < 18.5)	0.840	0.257–2.747	0.773	0.797	0.253–2.510	0.697	0.860	0.261–2.836	0.803
**Overweight (23** **≤BMI** ** < 25)**	**0.551**	**0.309–0.985**	**0.045**	**0.550**	**0.311–0.971**	**0.040**	0.543	0.291–1.014	0.055
Obesity stage 1 (25 ≤ BMI < 30)	0.706	0.395–1.263	0.240	0.740	0.411–1.334	0.315	0.751	0.405–1.390	0.360
Obesity stage 2 (30 ≤ BMI < 35)	0.402	0.128–1.265	0.119	0.493	0.155–1.566	0.229	0.509	0.150–1.723	0.277
**Obesity stage 3 (BMI** **≥35)**	4.896	0.996–24.069	0.051	**6.731**	**1.259–35.996**	**0.026**	**5.928**	**1.057–33.232**	**0.043**

Model 1: adjusted sex, age.

Model 2: adjusted sex, age, tinnitus, perceived stress.

Reference category (group): no dizziness (control).

BMI, body mass index.

Statistically significant variables are indicated in bold.

## Discussion

Using data from the 2019–2020 KNHANES for individuals aged 40 years or older, we have demonstrated that the weighted prevalence of chronic dizziness in the past 12 months is 4.8%. Moreover, we have found associations of chronic dizziness with old age, female sex, tinnitus, and high stress (12, 13), which have previously been reported for non-vestibular disease and specific vestibular disorders, including benign paroxysmal positional vertigo (14), Méniére's disease (15), and vestibular migraine (16). The novelty of our study lies in the investigation of the prevalence of chronic dizziness in the general population and its relationship with BMI. Previous studies of dizziness epidemiology did not distinguish between acute and chronic. The present study defined chronic dizziness as one or >3 months in a recent year. Our study found that 4.8% of all patients with dizziness experienced chronic dizziness for at least 3 months in the past year. Chronic dizziness, like other chronic conditions, including heart disease, cancer, and diabetes, can lead to ongoing medical attention or limit activities of daily living, or both (17). Our study found that 40.8% of patients with chronic dizziness experienced imbalance, such as standing difficulty, walking difficulty, and falling, which could be a sign of balance problem due to vestibular or neurological dysfunction. These data are consistent with the previous data on high falls or fall-related injuries in patients with dizziness (18). In the clinic, patients with chronic symptoms of dizziness and unsteadiness describe their problems in several ways. We believe that there are three types of history, such as the patient who started with one or more attacks of rotational vertigo, the patient with a progressive history of disequilibrium, and the patient with neither of these. In our study, the proportion of the patients with neither of these, that is, only the chronic dizziness group, was 3.0% of the total population and 59.2% of the chronic dizziness population (Table 2). This indicates that more than half of the patients with chronic dizziness complain of only subjective dizziness. This may explain why we have several patients with chronic dizziness whose thorough vestibular evaluations are unremarkable in the clinical field. Therefore, indiscriminate inspection using laboratory vestibular function tests does not help manage patients with chronic dizziness. It is crucial to determine the factors leading to chronic dizziness or imbalance to manage patients with dizziness considering that the causes of imbalance are multifactorial, including vestibular and non-vestibular components. In the general population, the reported prevalence of dizziness is diverse among studies, with a range of 17–30% (19), and it has been higher in the older adult population (20–23). In Korea, the prevalence of dizziness in this study (2019–2020) was higher than that in the previous study (25.3 vs. 16.7%), which supports that attention to dizziness is necessary (24).

In this study, BMI is a health factor that deserves recognition. The influence of BMI on clinical significance in patients with dizziness or imbalance remains controversial (18, 25–29), which may be related to the between-study heterogeneity, such as timing and duration of dizziness, postural instability, and coexistence of imbalance. In the Jackson Heart Study, 1,314 African Americans reporting dizziness demonstrated higher BMI, hypertension, and diabetes prevalence. However, higher BMI has not remained in adjusted regression analysis (25). A modified timed up and go test and computerized dynamic posturography showed that patients with postural imbalance had a higher risk of falling if they had obesity (BMI > 30 kg/m^2^) (30). An increase in body weight is correlated with greater balance instability and poor vestibular function (27, 31, 32), which is linked to falling risk in older adults (33). However, in our study, the chronic dizziness group had a lower BMI than the control group, which is consistent with the results of a previous German cohort study (26). Our study showed that overweight and mild obesity had significantly lower odds of chronic dizziness. This feature of the only dizziness group remained in the adjusted multivariate regression analysis (Table 5). Moreover, the imbalance group showed relatively higher odds in the obesity three group, similar to the previous studies on postural instability in the obese group (28). Obesity is a known risk factor for postural instability and imbalance. However, paradoxically, overweight and mild obesity confer an advantage in patients with only chronic dizziness. These findings align with the “obesity paradox” (34). The “obesity paradox” is the finding that obesity, which is a risk factor for developing chronic diseases, such as chronic kidney disease, heart disease, and diabetes mellitus, “counterintuitively,” is protective of mortality in those same chronic conditions (35–37). Marked obesity has adverse effects on health and longevity; however, the impact of being mild to moderately overweight is less specific (38, 39). Likewise morbid obesity is a definite risk factor of postural instability or fall, but the overweight or mild obesity can be helpful to human balance (28). One assumption is that the overweight might shift the body's center of gravity, bringing it closer to the ground, which could increase the sense of stability. In addition, excess body fat, increased metabolic reserve, and muscle strength might confer a protective benefit with respect to tolerating acute illness, stress and other medications like cardiovascular disease (40). In previous animal research, the bilateral vestibular lesion significantly reduced body weight, total fat mass, total muscle mass and adipocyte size enhanced by high sucrose and high fat diet but did not affect caloric intake with or without high sucrose and high fat diet (41). The authors suggested the vestibular system may contribute to the regulation of set points under excess energy conditions (42). Although we did not confirm the vestibular dysfunction in our group with chronic dizziness, some patients would have vestibular dysfunction, which could explain the decreased BMI in patients with chronic dizziness than in the control group. However, we could not judge whether the decreased BMI of chronic dizziness is the cause or result due to the limitation of study design of cross-sectional study. For confirmation, cohort study is needed. Therefore, we need to study the exact mechanism of alleviating the effect and optimal range of BMI in patients with dizziness with various conditions.

This study had several limitations. First, the clear causal and temporal relationships between chronic dizziness/imbalance and other related factors are difficult to verify in a cross-sectional study. Second, we could not evaluate each system's involvement in maintaining balance, such as peripheral and central sensory functions, musculoskeletal insufficiencies, and deficits in cognitive postural control, particularly in the vestibular system. Third, although some advantages of using BMI to measure obesity are that it is inexpensive and allows risk stratification, BMI is not a reliable measurement of obesity because it measures total body mass, not only adipose tissue (40, 43). The obvious paradox may be due to BMI. Therefore, further validation study is needed.

## Data availability statement

The raw data supporting the conclusions of this article will be made available by the authors, without undue reservation.

## Ethics statement

The studies involving human participants were reviewed and approved by Institutional Review Board (IRB) of the Korean Centers for Disease Control and Prevention (KCDC) [IRB: 2018-01-03-C-A and 2018-01-03-2C-A]. The patients/participants provided their written informed consent to participate in this study.

## Author contributions

EKi and H-JS acquired, analyzed the data, and drafted the manuscript. HL and EKw acquired, analyzed the data, and revised the manuscript. S-HJ conceptualized, supervised the study, and revised the manuscript. All authors contributed to the article and approved the submitted version.

## Funding

This work was supported by Chungnam National University Hospital Research Fund, 2021 (2021-CF-028) and Chungnam National University Fund (2019-0147-02).

## Conflict of interest

The authors declare that the research was conducted in the absence of any commercial or financial relationships that could be construed as a potential conflict of interest.

## Publisher's note

All claims expressed in this article are solely those of the authors and do not necessarily represent those of their affiliated organizations, or those of the publisher, the editors and the reviewers. Any product that may be evaluated in this article, or claim that may be made by its manufacturer, is not guaranteed or endorsed by the publisher.
