# Supplementary material for: One-year prevalence and clinical characteristics in chronic dizziness: The 2019–2020 Korean National Health and Nutrition Examination Survey
Source: Front Neurol. 2022 Dec 1;13:1016718. doi: 10.3389/fneur.2022.1016718 (PMC9751592; doi:10.3389/fneur.2022.1016718)
Supplement: Supplementary file 1 [file Table_1.docx]

**Appendix**. Nutrition factor for chronic dizziness.

| **Variable** | **Number**  **(unweighted/weighted)** | **Chronic**  **dizziness** | **Number**  **(unweighted/weighted)** | **No dizziness**  **(control)** | **p** |
| --- | --- | --- | --- | --- | --- |
| *nutrition intake test* |  |  |  |  |  |
| Water intake (g) (mean) | 299/1,328,314 | 873.8 | 3,740/20,734,060 | 1020.7 | **0.006** |
| Food intake (g) (mean) | 299/1,328,314 | 1321.4 | 3,740/20,734,060 | 1553.0 | **< 0.001** |
| Carbohydrate intake (g) (mean) | 299/1,328,314 | 249.5 | 3,740/20,734,060 | 278.8 | **0.001** |
| Protein intake (g) (mean) | 299/1,328,314 | 55.5 | 3,740/20,734,060 | 68.6 | **< 0.001** |
| Fat intake (g) (mean) | 299/1,328,314 | 32.3 | 3,740/20,734,060 | 42.6 | **< 0.001** |
| Dietary fiber intake (g) (mean) | 299/1,328,314 | 23.4 | 3,740/20,734,060 | 26.5 | **0.004** |
| Ca intake (mg) (mean) | 299/1,328,314 | 452.8 | 3,740/20,734,060 | 504.1 | **0.020** |
| P intake (mg) (mean) | 299/1,328,314 | 877.1 | 3,740/20,734,060 | 1040.8 | **< 0.001** |
| Fe intake (mg) (mean) | 299/1,328,314 | 9.5 | 3,740/20,734,060 | 11.5 | **< 0.001** |
| Na intake (mg) (mean) | 299/1,328,314 | 2722.1 | 3,740/20,734,060 | 3390.0 | **< 0.001** |
| K intake (mg) (mean) | 299/1,328,314 | 2531.3 | 3,740/20,734,060 | 2934.8 | **< 0.001** |
| Vitamin A(retinol) intake(ugRAE) (mean) | 299/1,328,314 | 345.1 | 3,740/20,734,060 | 379.6 | 0.365 |
| ß carotene intake (ug) (mean) | 299/1,328,314 | 2394.3 | 3,740/20,734,060 | 2922.3 | **0.002** |
| Vitamin B_1_(thiamine) intake (mg) (mean) | 299/1,328,314 | 1.1 | 3,740/20,734,060 | 1.3 | **< 0.001** |
| Vitamin B_2_(riboflavin) intake (mg) (mean) | 299/1,328,314 | 1.3 | 3,740/20,734,060 | 1.6 | **< 0.001** |
| Vitamin B_3_(niacin) intake (mg) (mean) | 299/1,328,314 | 10.4 | 3,740/20,734,060 | 12.4 | **< 0.001** |
| Folic acid intake (ugDFE) (mean) | 299/1,328,314 | 288.3 | 3,740/20,734,060 | 336.5 | **< 0.001** |
| Vitamin C intake (mg) (mean) | 299/1,328,314 | 56.3 | 3,740/20,734,060 | 66.7 | **0.027** |
| Carbohydrate intake (%) |  |  |  |  | **0.040** |
| < RNI | 24/108,744 | 8.2 | 182/976,540 | 4.7 |  |
| ≥ RNI | 275/1,219,569 | 91.8 | 3,558/19,757,519 | 95.3 |  |
| Protein intake (%) |  |  |  |  | **< 0.001** |
| < RNI | 202/853,675 | 64.3 | 1,951/9,965,730 | 48.1 |  |
| ≥ RNI | 97/474,639 | 35.7 | 1,789/10,768,329 | 51.9 |  |
| Dietary fiber intake (%) |  |  |  |  | **0.003** |
| < AI | 145/625,901 | 47.1 | 1,378/7,540,468 | 36.4 |  |
| ≥ AI | 154/702,412 | 52.9 | 2,362/13,193,591 | 63.6 |  |
| Ca intake (%) |  |  |  |  | 0.371 |
| < RNI | 274/1,208,528 | 91.0 | 3,300/18,239,658 | 88.0 |  |
| RNI ≤ < UL | 24/114,969 | 8.7 | 432/2,432,056 | 11.7 |  |
| ≥ UL | 1/4,816 | 0.4 | 8/62,347 | 0.3 |  |
| P intake (%) |  |  |  |  | **< 0.001** |
| < RNI | 127/556,332 | 41.9 | 1,026/5,051,348 | 24.4 |  |
| RNI ≤ < UL | 172/771,981 | 58.1 | 2,711/15,668,022 | 75.6 |  |
| ≥ UL |  |  | 3/14,688 | 0.1 |  |
| Fe intake (%) |  |  |  |  | **0.001** |
| < RNI | 196/860,176 | 64.8 | 1,987/10,620,809 | 51.2 |  |
| RNI ≤ < UL | 102/463,320 | 34.9 | 1,738/10,020,316 | 48.3 |  |
| ≥ UL | 1/4,816 | 0.4 | 15/92,934 | 0.4 |  |
| Na intake (%) |  |  |  |  | **< 0.001** |
| < AI | 65/281,785 | 21.2 | 465/2,412,086 | 11.6 |  |
| ≥ AI | 234/1,046,529 | 78.8 | 3,275/18,321,974 | 88.4 |  |
| K intake (%) |  |  |  |  | **0.005** |
| < AI | 249/1,083,323 | 81.6 | 2,757/14,915,768 | 71.9 |  |
| ≥ AI | 50/244,990 | 18.4 | 983/5,815,292 | 28.1 |  |
| Vitamin A(retinol) intake (%) |  |  |  |  | 0.224 |
| < RNI | 272/1,212,958 | 91.3 | 3,400/18,772,522 | 90.5 |  |
| RNI ≤ < UL | 25/105,978 | 8.0 | 332/1,917,625 | 9.2 |  |
| ≥ UL | 2/9,377 | 0.7 | 8/43,912 | 0.2 |  |
| Vitamin B_1_(thiamine) intake (%) |  |  |  |  | **0.001** |
| < RNI | 204/892,206 | 67.2 | 2,170/11,436,939 | 55.2 |  |
| ≥ RNI | 95/436,108 | 32.8 | 1,570/9,297,120 | 44.8 |  |
| Vitamin B_2_(riboflavin) intake (%) |  |  |  |  | **0.001** |
| < RNI | 208/858,206 | 64.6 | 2,099/10,792,502 | 52.1 |  |
| ≥ RNI | 91/470,107 | 35.4 | 1,641/9,941,558 | 47.9 |  |
| Vitamin B_3_(niacin) intake (%) |  |  |  |  | **0.021** |
| < RNI | 256/1,124,949 | 84.7 | 3,023/16,164,675 | 78.0 |  |
| ≥ RNI | 43/203,365 | 15.3 | 717/4,569,384 | 22.0 |  |
| Folic acid intake (%) |  |  |  |  | 0.130 |
| < RNI | 236/1,036,643 | 78.0 | 2,696/14,876,761 | 71.8 |  |
| RNI ≤ < UL | 62/287,913 | 21.7 | 1,028/5,791,012 | 27.9 |  |
| ≥ UL | 1/3,757 | 0.3 | 16/66,285 | 0.3 |  |
| Vitamin C intake (%) |  |  |  |  | 0.341 |
| < RNI | 259/1,135,666 | 85.5 | 3,090/17,130,134 | 82.6 |  |
| ≥ RNI | 40/192,648 | 14.5 | 650/3603,925 | 17.4 |  |
| RNI (Recommended Nutrient Intake) |  |  |  |  |  |
| AI (Adequate Intake) |  |  |  |  |  |
| UL (Tolerable upper intake level) |  |  |  |  |  |
